# Unpacking brown food‐webs: Animal trophic identity reflects rampant microbivory

**DOI:** 10.1002/ece3.2951

**Published:** 2017-04-09

**Authors:** Shawn A. Steffan, Yoshito Chikaraishi, Prarthana S. Dharampal, Jonathan N. Pauli, Christelle Guédot, Naohiko Ohkouchi

**Affiliations:** ^1^ Department of EntomologyUniversity of WisconsinMadisonWIUSA; ^2^ US Department of AgricultureAgricultural Research ServiceMadisonWIUSA; ^3^ Department of BiogeochemistryJapan Agency for Marine Science & TechnologyYokosukaJapan; ^4^ Institute of Low Temperature ScienceHokkaido UniversitySapporoJapan; ^5^ Department of Forest & Wildlife EcologyUniversity of WisconsinMadisonWIUSA

**Keywords:** detritivory, detritus, food chain, microbe, microbiome, omnivore

## Abstract

Detritivory is the dominant trophic paradigm in most terrestrial, aquatic, and marine ecosystems, yet accurate measurement of consumer trophic position within detrital (=“brown”) food webs has remained unresolved. Measurement of detritivore trophic position is complicated by the fact that detritus is suffused with microbes, creating a detrital complex of living and nonliving biomass. Given that microbes and metazoans are trophic analogues of each other, animals feeding on detrital complexes are ingesting other detritivores (microbes), which should elevate metazoan trophic position and should be rampant within brown food webs. We tested these hypotheses using isotopic (^15^N) analyses of amino acids extracted from wild and laboratory‐cultured consumers. Vertebrate (fish) and invertebrate detritivores (beetles and moths) were reared on detritus, with and without microbial colonization. In the field, detritivorous animal specimens were collected and analyzed to compare trophic identities among laboratory‐reared and free‐roaming detritivores. When colonized by bacteria or fungi, the trophic positions of detrital complexes increased significantly over time. The magnitude of trophic inflation was mediated by the extent of microbial consumption of detrital substrates. When detrital complexes were fed to vertebrate and invertebrate animals, the consumers registered similar degrees of trophic inflation, albeit one trophic level higher than their diets. The wild‐collected detritivore fauna in our study exhibited significantly elevated trophic positions. Our findings suggest that the trophic positions of detrital complexes rise predictably as microbes convert nonliving organic matter into living microbial biomass. Animals consuming such detrital complexes exhibit similar trophic inflation, directly attributable to the assimilation of microbe‐derived amino acids. Our data demonstrate that detritivorous microbes elevate metazoan trophic position, suggesting that detritivory among animals is, functionally, omnivory. By quantifying the impacts of microbivory on the trophic positions of detritivorous animals and then tracking how these effects propagate “up” food chains, we reveal the degree to which microbes influence consumer groups within trophic hierarchies. The trophic inflation observed among our field‐collected fauna further suggests that microbial proteins represent an immense contribution to metazoan biomass. Collectively, these findings provide an empirical basis to interpret detritivore trophic identity, and further illuminate the magnitude of microbial contributions to food webs.

## 1 Introduction

The majority of global primary productivity is not consumed as living tissues, but rather as nonliving detritus (Colinvaux, 1978; Moore et al., 2004; Polis & Strong, 1996). Biomass gets channeled by consumers primarily into “brown” food chains, or detrital pathways (Hagen et al., 2012; Hyodo, Matsumoto, Takematsu, & Itioka, 2014), as opposed to “green” ones (i.e., food chains supported by living autotrophic biomass). It is not surprising then, that detritivore biomass generally far exceeds that of herbivore or carnivore biomass (Hagen et al., 2012; Moore & de Ruiter, 2012; Polis & Strong, 1996). Despite large temporal and seasonal variability (Duarte & Cebrián, 1996; Moore & de Ruiter, 2012), detritivory can be considered the dominant trophic paradigm in most ecosystems (Coleman, 1996; Hagen et al., 2012). As the dominant consumer group on Earth, detritivores mediate the functioning of ecosystems, affecting organisms across the trophic spectrum (Bardgett & Cook, 1998; Brose & Scheu, 2014; Dharampal & Findlay, 2017; Hagen et al., 2012; Moore et al., 2004).

**Figure 1:**
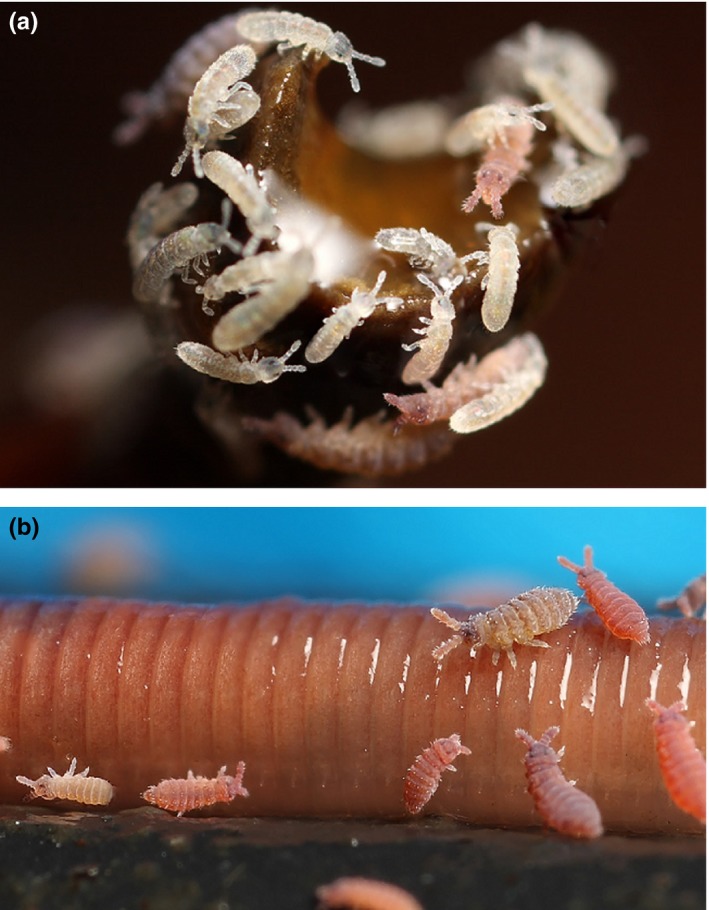
(a) Nymphal springtails (*Ceratophysella*) feeding on bacteria‐ and fungi‐colonized detritus. (b) Large and small detritivores: an earthworm (Lumbricidae) is visited by several tiny springtails (Hypogastruridae). Photographs courtesy: Brian Valentine

Importantly, it is the microbial community in a given system that tends to occupy the majority of detritivore biomass (Bardgett & Cook, 1998; Coleman, 1996; Moore & de Ruiter, 2012; Peterson & Luxton, 1982). As microbes consume a detrital resource, they often become enmeshed in the detritus forming a detrital complex. In brown food chains, this heterotrophic biomass flowing directionally to “higher” metazoan consumers is mostly bacterial, protozoan, and/or fungal in origin (Bardgett & Cook, 1998; Bengtsson, Setala, & Zheng, 1996; Crotty, Blackshaw, & Murray, 2011; Fenchel, 1992; Hagen et al., 2012; Hall & Meyer, 1998; Pollierer, Dyckmans, Scheu, & Haubert, 2012). As the detrital complex is comprised of both living and nonliving elements (Coleman, 1996; Moore, Walter, & Hunt, 1988), metazoans in brown food webs, such as earthworms or springtails (Figure 1), likely consume not only decaying matter but also the microorganisms suffused throughout it (Bardgett & Cook, 1998; Briones, Garnett, & Piearce, 2005; Digel, Curtsdotter, Riede, Klarner, & Brose, 2014; Peterson & Luxton, 1982). If detritivorous microbes represent the largest and most basal prey group in most food webs, their trophic roles should strongly influence the trophic functions (i.e., trophic positions) of the resident metazoan detritivores. More specifically, the disproportionately high availability of microbial prey from brown resource pools (compared to herbivorous prey from green food chains) can be expected to exert greater influence on the trophic positions of higher‐order consumers (carnivores) that often feed opportunistically from both brown and green food webs. Thus, microbes are not only foundational as prey, but also as drivers of carnivore trophic identity and heterotrophic productivity (Digel et al., 2014; Fitter et al., 2005; van der Heijden, Bardgett, & van Straalen, 2008).

As metazoan detritivores may not readily separate microbes from their detrital substrate, pure detritivory (diets devoid of microbial protein) among metazoan consumers is likely rare in nature. The assessment of animal trophic position therefore needs to accommodate the fact that detritus is a heterogeneous complex and trophically dynamic, which would provide varying proportions of detrital versus microbial protein when consumed en masse by meso‐ or macrofauna (Brose & Scheu, 2014; Moore & de Ruiter, 2012; Moore et al., 2004). As these fauna consume significant quantities of microbe‐suffused plant detritus, they consume fellow heterotrophs. It has long been thought that when animals consume microbes, the interaction represents a predator–prey relationship (Bardgett & Cook, 1998; Coleman, 1996; Digel et al., 2014). Recent work has shown that consumption of heterotrophic microbes registers as carnivory, meaning that macro‐ and microbiota are trophic analogues of one another, and can be interdigitated within a food chain (Steffan et al., 2015). Because detrital complexes are generally comprised of multiple heterotrophic groups (Bengtsson et al., 1996; Brose & Scheu, 2014; Moore et al., 1988), the consumption of detrital complexes constitutes intraguild predation (Digel et al., 2014), which should inflate the consumer's trophic position. Over time, the average trophic position of a given detrital mass can be expected to increase as the organisms therein consume the resource, as well as one another. Thus, any given mass of detritus may represent a reticulate food web microcosm (Digel et al., 2014).

For the meso‐ and macrofauna feeding on such detrital complexes, microbivory will be rampant and should measurably elevate the trophic positions of the fauna. While the microbial decomposition of detritus has been evidenced via ^15^N enrichment in soil communities (Hyodo et al., 2008; Ponsard & Arditi, 2000; Tayasu & Hyodo, 2010), the heterogeneous and dynamic nature of the detrital complex can complicate the interpretation of the trophic positions of microbes and detritivorous metazoans. This issue may be distilled into a few basic questions: How do microbes shape the trophic identities of metazoans in brown food webs? What is the trophic position of a given detrital mass at a given point in time? And finally, what are the trophic positions of detritivores feeding on such detritus? To adequately address the issue of detritivore trophic identity, a more comprehensive trophic framework—one that includes microbes within an empirically derived trophic hierarchy—will be needed.

Here, we address the question of detritivore trophic identity using an array of wild and laboratory‐cultured metazoan and microbial consumers fed plant‐, animal‐, and detritus‐based diets. Along with the traditional bulk ^15^N technique, we used compound‐specific isotopic analysis, which allowed precise and accurate estimates of the trophic positions of detrital complexes at multiple stages of microbial decay, as well as the trophic positions of animals feeding on the detritus or detrital complexes. We tested the following hypotheses: (1) Microbial consumption of detritus elevates the trophic position of detrital complexes; (2) mechanistically, microbe‐mediated trophic inflation is a direct function of the degree to which microbial consumers have converted detrital amino acids into microbial amino acids; (3) consumers of a detrital complex register one trophic level above the complex. We characterize the foundational trophic roles of microbes, explain the high ^15^N signals often observed among detritivorous fauna (Chahartaghi, Langel, Scheu, & Ruess, 2005; Hyodo et al., 2008, 2014; Maraun et al., 2011; Miller et al., 2012; Okuzaki, Tayasu, Okuda, & Sota, 2009), and show that a broad diversity of field‐collected detritivores closely mirrored the trophic patterns observed among our laboratory‐reared detritivores.

## 2 Materials and Methods

### 2.1 Culturing of organisms

The detritivore species in this study were each fed a single diet of known isotopic composition, and these diets were provisioned based on four basic protocols. First, any given detrital mass was oven‐dried at 45°C in advance of the experiment, thereby halting all microbial activity and holding constant the trophic position of the detritus. Second, all detrital complexes were homogenized into a fine‐grain blend, which would ensure that the detritivores in our controlled‐feeding study would consume all components of the homogenate (rather than feeding preferentially on subsets of any given detrital complex). Third, we chose animal consumer species that all shared a key attribute: they promptly consumed their daily provision of the diet. Fourth, we reared the consumers from a very early stage in their ontogeny, ensuring that their isotopic composition derived almost exclusively from the diet we provided. Samples of consumers were curated (freezing, drying, maceration of whole organism), and aliquots (1.5–2.5 mg) were submitted for isotopic analysis.

The plant‐based homogenate consisted of dry soy powder and wheat flour, as formulated in previous work (Steffan et al., 2015) and was used exclusively to culture pantry moths (*Plodia interpunctella* Hubner), “enoki” fungi (*Flammulina velutipes* [Curtis]), and the aerobic rhizobacterium, *Bacillus subtilis* Ehrenberg. Before initiating any given culture, samples (10 mg) of the soy–wheat homogenate were collected, dried further, weighed, and placed in labeled vials for subsequent isotopic analysis. Larvae of *P. interpunctella* were cultured within 100 ml glass jars. In each jar, 20–30 larvae were allowed to develop through pupation entirely on the dry soy–wheat homogenate. At pupation, individual specimens of *P. interpunctella* were frozen, dried, weighed, and placed in labeled vials for subsequent isotopic analysis.

Within each of six 100 ml glass jars, the fungus‐ or bacterium‐colonized soy–wheat cultures were created by first autoclaving (121°C, 15 min) glass jars (250 ml) containing a moistened soy–wheat homogenate (25 ml deionized water per 100 mg homogenate). After cooling, the substrate was inoculated with either *F. velutipes* mycelia or *B. subtilis* spores. Microbial cultures were allowed to incubate at 30°C for 3–4 weeks before being dried and homogenized. All jars of fungal cultures were blended into a single homogenate, and all jars of the bacterial cultures were blended into a single homogenate. Each homogenate was parsed out among six new 100 ml glass jars, such that there were six jars containing the *F. velutipes* homogenate, and six jars of the *B. subtilis* homogenate. Samples of the homogenate in each jar (10 mg) were collected, dried, weighed, and placed in vials for subsequent isotopic analyses.

Within each of six 100 ml glass jars, 20 larvae of *P. interpunctella* larvae were allowed to develop entirely on the detrital homogenate created from the *F. velutipes* cultures. Similarly, 20 larvae of the red flour beetle (*Tribolium castaneum* Herbst) were allowed to develop entirely on the detrital homogenate created from the *B. subtilis* culture. At pupation, individual specimens of *P. interpunctella* and *T. castaneum* were frozen, dried, weighed, and placed in labeled vials for subsequent isotopic analysis.

Frozen, dried cadavers of larval *Spodoptera frugiperda* (strict herbivores) were homogenized and fed to the common guppy, *Poecilia reticulata*, using established rearing protocols (Steffan et al., 2015). Very young fry (8–9 mm long) were used, and the *S. frugiperda* homogenate was their sole food source; further, the parents of these fry were fed the same diet for 3–4 weeks prior to the live birth of the fry. A separate group of *P. reticulata* fry were fed dried, homogenized cadavers of *S. frugiperda* that had been killed and largely consumed by the entomopathogenic fungus, *Beauveria bassiana*. Again, only very young fry (8–9 mm) were cultured on this fungus‐colonized carrion, following 3–4 weeks of feeding this diet to the parents of the fry. All fry were fed for 7–8 weeks, representing greater than a 10‐fold increase in weight, until they had reached sexual maturity.

Sterile detrital substrates consisting of either potato dextrose agar (PDA) or yeast extract maltose agar (YEMA) were autoclaved and poured into petri dishes. PDA plates were inoculated with *F. velutipes* (*N* = 12), and YEMA plates were inoculated with the bacterium, *Streptomyces* (*N* = 12). Noninoculated control plates were incubated in‐parallel. Plates were incubated at 30°C for 0, 7, and 14 days. At each of the three time steps (0, 7, and 14 days), four plates from the fungal and four from the bacterial cultures were photographed to quantify % coverage of the substrate. The photographed plates were then frozen, lyophilized, and homogenized for isotopic analyses. All isotopic measurements were conducted using a 4.0–4.5 mg aliquot drawn from the entire contents of any given plate.

### 2.2 Collection of wild specimens

Fruit flies (*Drosophila suzukii*) were collected at five agricultural sites in southern Wisconsin (Dane, Sauk, and Richland Counties), USA. Waterpan traps were set within farm sites (raspberry plantings), as well as along bordering woodland habitats (mixed deciduous and coniferous forests). Specimens were collected in July, August, October, and November of 2014. Specimens were curated first in 70% ethanol and then lyophilized for 2 days. At each site, 15 specimens were randomly selected from the pool of fly specimens collected on a given date. A total of 10 samples were prepared for isotopic analysis. The wings from each fly were excised, ensuring not to include any soft tissues (confining samples to wings precluded the issue of rapid microbial degradation in soft tissues while flies were in waterpan traps). Bark beetles (Scolytinae: Curculionidae) were collected in 2014 as they emerged from pine (*Pinus* sp.) and ash (*Fraxinus* sp.) tree cuttings in south‐central Wisconsin. Adult beetle specimens were curated first in 70% ethanol and then lyophilized for 2 days. Each sample represented a single bark beetle. A total of three beetle samples were prepared for isotopic analysis. Earthworms (Lumbricidae) were collected from moist soil in forest understories at the University of Wisconsin campus in Madison, Wisconsin, USA. Springtails (families Hypogastruridae, Isotomidae, and Sminthuridae) were collected in waterpan traps set within cranberry marshes in central Wisconsin (Wood Co.). Specimens were curated first in 70% ethanol and then lyophilized for 2 days.

### 2.3 Amino acid isotopic analysis

The nitrogen (^15^N) isotopic compositions of amino acids (i.e., glutamic acid, phenylalanine) were analyzed using established protocols (Chikaraishi, Kashiyama, Ogawa, Kitazato, & Ohkouchi, 2007; Chikaraishi et al., 2009) at the Department of Biogeochemistry, Japan Institute of Marine‐Earth Science and Technology, Yokosuka, Japan. Specimens were hydrolyzed, and the hydrolysate was washed with *n*‐hexane/dichloromethane. Derivatization of amino acids was performed sequentially with thionyl chloride/2‐propanol (1/4) and pivaloyl chloride/dichloromethane (1/4). Gas chromatography (GC) using a 6890N GC connected to a flame ionization detector and nitrogen–phosphorus detector was used to determine amino acid abundance. The Pv/iPr derivatives of amino acids were injected using a programmable temperature vaporizing injector (Gerstel) into a VF‐35 ms capillary column (Agilent Technologies). Stable nitrogen isotopic composition of amino acids was determined by GC‐combustion‐isotope ratio mass spectrometry (GC/C/IRMS) using a 6890N GC (Agilent Technologies) instrument coupled to a Delta^plus^XP IRMS instrument through combustion (950°C) and reduction (550°C) furnaces, countercurrent dryer (Permeable membrane, Nafion™), and liquid nitrogen CO_2_ trap via a GC‐C/TC III interface (Thermo Fisher Scientific). Further details of the GC/C/IRMS analyses are available within Appendix S1.

### 2.4 Trophic position computations

Estimation of trophic position (TP_glu‐phe_) was based on the isotopic ratios of two specific amino acids: glutamic acid (glu) and phenylalanine (phe). The ^15^N values of glutamic acid (δ^15^N_glu_) and phenylalanine (δ^15^N_phe_) were substituted into the following equation (Chikaraishi et al., 2009):

$$\text{TP}=\frac{\delta^{15}N_{\mathrm{glu}}-\delta^{15}N_{\mathrm{phe}}+\beta }{\Delta_{\mathrm{glu}-\mathrm{phe}}}+\lambda ,$$

where β (~8.4‰) corrects for the difference in ^15^N values between glutamic acid and phenylalanine in C_3_ plants, Δ_glu‐phe_ represents the net trophic discrimination between glutamic acid and phenylalanine, and λ represents the basal trophic level (=1) of the food web. Net trophic discrimination (Δ_glu‐phe_) was calculated as the difference in glutamic acid and phenylalanine values between a consumer and its diet: Δ_glu‐phe_ = (δ^15^N_consumer_ − δ^15^N_diet_)_glu_ − (δ^15^N_consumer_ − δ^15^N_diet_)_phe_ (Chikaraishi et al., 2009). Trophic position estimation based on compound‐specific stable isotope analysis has demonstrated unprecedented accuracy and precision, making such approaches preferable to bulk‐N analysis (Steffan, Chikaraishi, et al., 2013).

### 2.5 Statistical analyses

Linear regression analyses were used to characterize the relationships between (1) time and % consumption of detrital plates, (2) δ^15^N_glu_ and % consumption, (3) δ^15^N_phe_ and % consumption, and (4) δ^15^N_bulk_ and % consumption. One‐way ANOVA followed by post hoc pairwise contrasts were used to distinguish among trophic groups within “green” and “brown” food chains. One‐sample *t* tests were used to test whether the mean trophic position of a given consumer group was different than its nearest integer trophic position.

## 3 Results

### 3.1 Green food chain

**Figure 2:**
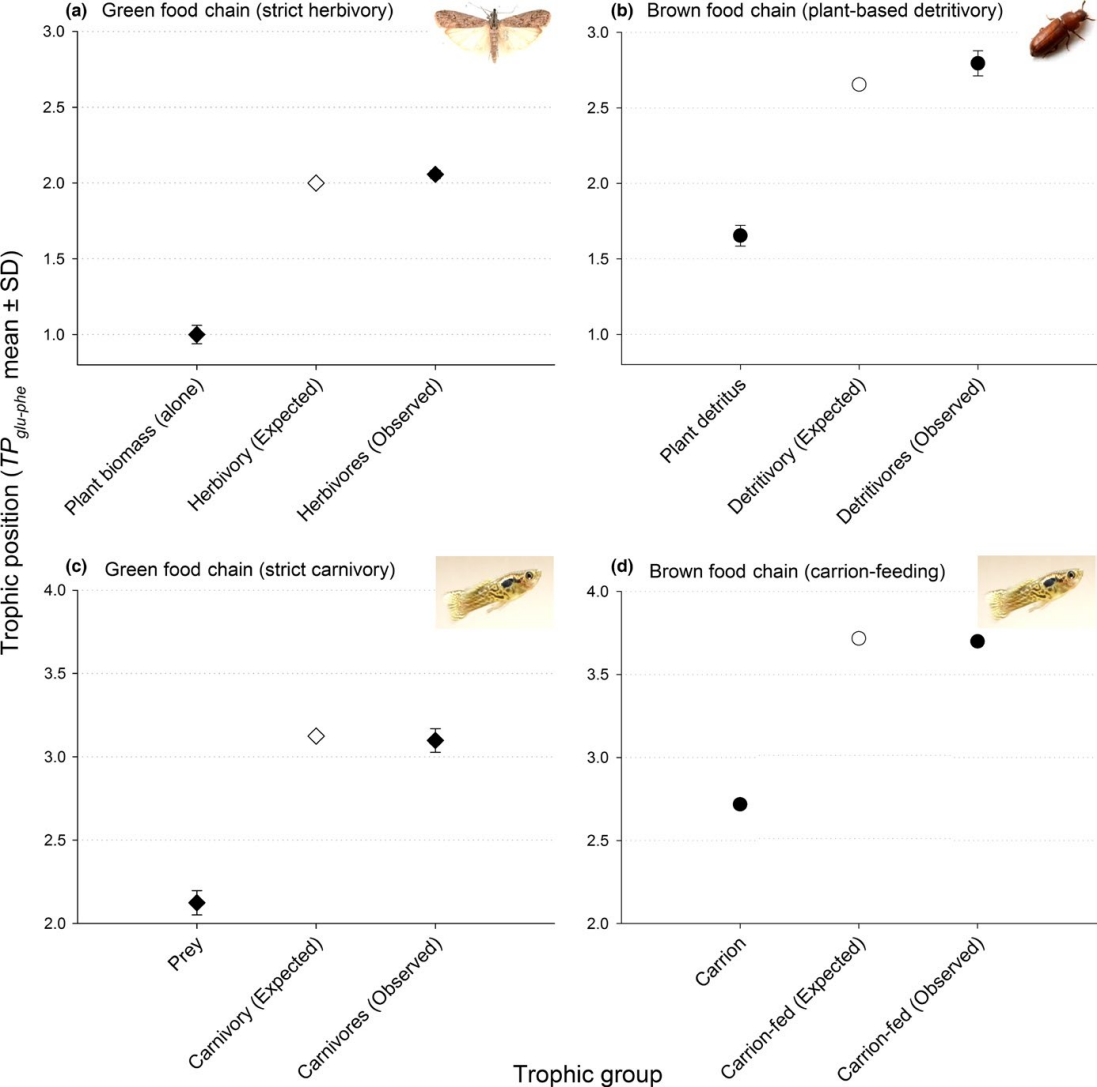
(a) Mean trophic positions (±σ) of a soy–wheat homogenate, and the consumer of this homogenate: pantry moths, *Plodia interpunctella*. (b) Mean trophic positions of microbe‐colonized detritus and the consumers of this detritus: red flour beetles, *Tribolium castaneum*, and pantry moths, *P. interpunctella*. (c) Mean trophic positions of armyworms, *Spodoptera frugiperda* (strict herbivores), and the consumer of an armyworm homogenate: common guppies, *Poecilia reticulata*. (d) Mean trophic position of microbe‐colonized animal detritus (carrion); mean trophic position of the guppy, *P. reticulata*, cultured exclusively on the carrion

Plant biomass alone (no microbial activity) registered a mean trophic position of 1.00 ± 0.0153 (mean ± *SE*), corresponding closely to the standard trophic position for plants (TP ~ 1) in any given food chain (see Appendix S1 datasets for all raw data). The herbivorous flour moths that had been cultured exclusively on plant biomass (i.e., strict herbivores) registered at 2.06 ± 0.035 (Figure 2a). This trophic position was not significantly different from TP ~ 2 (*t*_2_ = 1.61, *p *=* *.249), but was significantly higher than the plant‐based diet, itself (*t*_4_ = −27.49, *p *<* *.001).

Herbivore biomass alone (no microbial activity) registered a mean trophic position of 2.12 ± 0.042 (mean ± *SE*), corresponding closely to the standard trophic position for herbivores (TP ~ 2). The carnivores (guppies) that had been reared exclusively on herbivorous caterpillars registered approximately one trophic level higher than their prey (Figure 2c), at trophic position 3.10 ± 0.041. Caterpillar trophic position (the diet) was not dissimilar from the expected TP ~ 2 (*t*_2_ = 2.94, *p *=* *.099), and the guppies were not dissimilar from the expected TP ~ 3 (*t*_2_ = 2.41, *p *=* *.137). Carnivore trophic position was significantly higher than that of their diet (*t*_4_ = −16.64, *p *<* *.001).

### 3.2 Brown food chain

Plant detritus colonized by the fungus, *F. velutipes*, produced a trophic position of 1.71 ± 0.026, representing a 71% increase in trophic position. When colonized by the bacterium, *B. subtilis*, the detrital complex registered a trophic position of 1.60 ± 0.026, a 60% increase in trophic position. Collectively, the plant detritus colonized/consumed by microbes registered a mean trophic position of 1.65 ± 0.028 (Figure 2b), representing a significant departure from the no‐microbe plant biomass (*t *=* *13.38, *p *<* *.001). The microbe‐colonized detritus diverged significantly from both TP ~ 1 (*t*_5_ = 23.19, *p *<* *.001) and TP ~ 2 (*t*_5_ = −12.28, *p *<* *.001), placing it at a distinctly intermediate (noninteger) trophic position (Figure 2b). When animal cadavers (herbivores, trophic position 2.1) were consumed by fungi and bacteria, the trophic position of the carrion increased to 2.7 (again, an increase of 0.60 trophic levels).

Consumer groups feeding exclusively on the microbe‐colonized plant detritus would be expected to register one trophic level higher, at or near TP ~ 2.65 (=TP ~ 1.65 + 1.00). The flour moth larvae in our study registered a mean trophic position of 2.80 ± 0.058 after being cultured exclusively on fungus‐colonized detritus. Red flour beetles registered at 2.79 ± 0.048 after feeding exclusively on bacteria‐colonized detritus. Altogether, the mean trophic position of consumers reared on microbe‐colonized detritus was 2.79 ± 0.034 (Figure 2b). While this trophic position represented a statistically significant departure from the expected TP ~ 2.65 (*t*_5_ = 4.30, *p *=* *.008), functionally, the difference was quite small.

Carrion (armyworm larvae, colonized/consumed by microbes) registered a mean trophic position of 2.72 ± 0.014. This trophic position was significantly greater than TP ~ 2 (*t*_2_ = −52.35, *p *<* *.001) and significantly less than TP ~ 3 (*t*_2_ = −20.69, *p *<* *.001), placing the carrion at an intermediate (noninteger) trophic position (Figure 2d). Consumers of this carrion would be expected to register one trophic level higher, at or near TP ~ 3.72 (=TP ~ 2.72 + 1.00). The carrion feeders in our study (guppies) registered a mean trophic position of 3.70 ± 0.012, which was virtually identical to the expected TP (*t*_2_ = −1.79, *p *=* *.216) while being significantly elevated above the carrion itself (*t*_4_ = −54.86, *p *<* *.001; Figure 2d).

### 3.3 Ontogeny of the detrital complex

**Figure 3:**
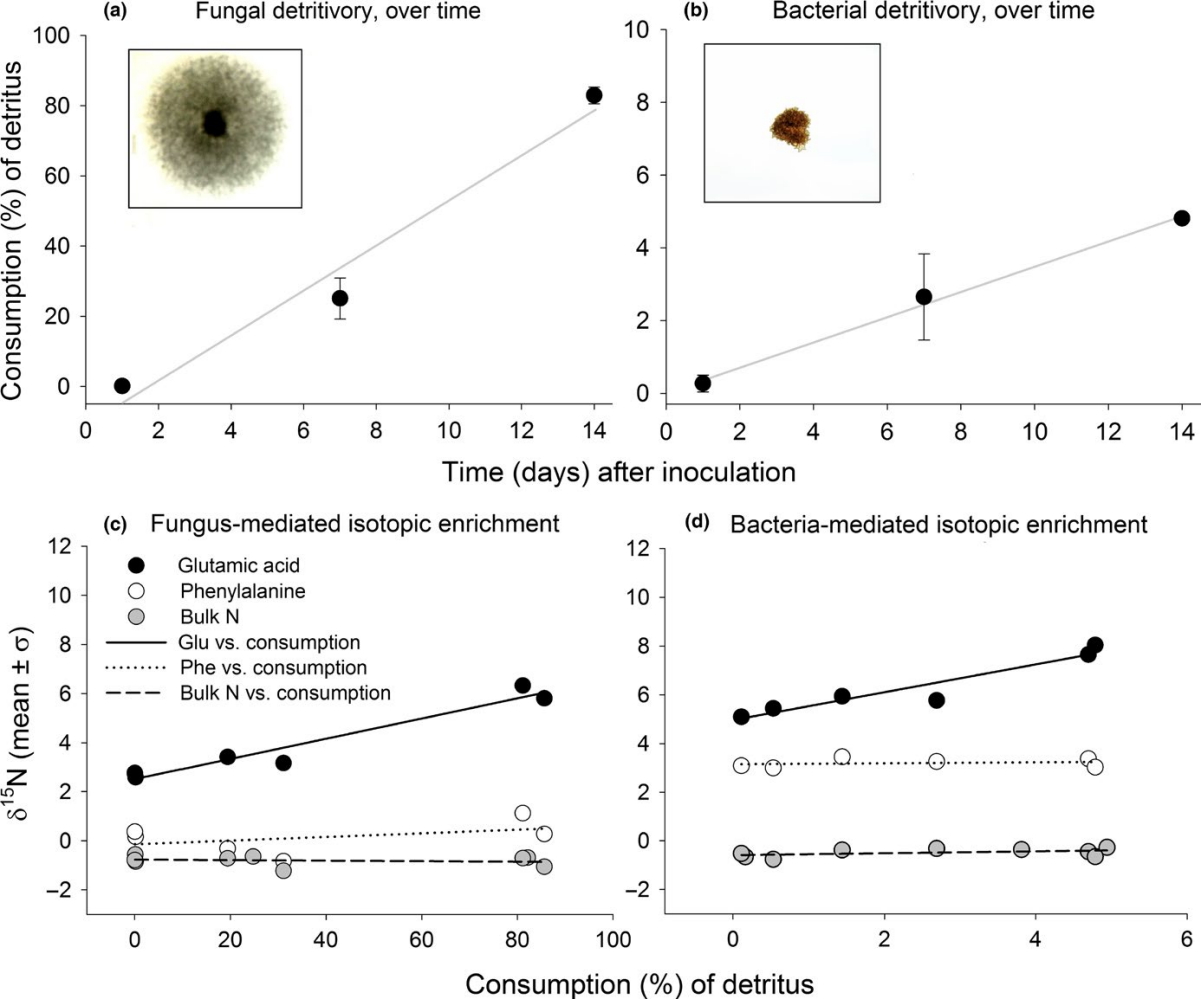
Mean consumption of a detrital substrate (measured as % coverage) by (a) the fungus, *Flammulina velutipes*, and (b) the filamentous bacterium, *Streptomyces*, at 0, 7, and 14 days. Across this consumption gradient, ^15^N signals of glutamic acid, phenylalanine, and bulk‐N in (c) cultures of the fungus *F. velutipes*, and (d) the bacterium, *Streptomyces*

The area (%) occupied by microbial growth within the agar media plates increased markedly over the course of 14 days (Figure 3a,b). The degree of consumption of this detrital resource was positively correlated with time for both the fungi and bacteria (Fungi linear regression: *y *=* *6.42*x* − 11.06, *R* ^2^ = .96, *F*_1,8_ = 170.23, *p *<* *.001; Bacteria linear regression: *y *=* *0.348*x* + 0.023, *R* ^2^ = .91, *F*_1,8_ = 69.27, *p *<* *.001). As the detrital resource was consumed by fungi, the ^15^N isotopic signal of glutamic acid in the detrital mass increased predictably with the degree of consumption (linear regression: *y *=* *0.041*x* + 2.51, *R* ^2^ = .94, *F*_1,5_ = 66.70; *p *=* *.001; Figure 3c). Conversely, the isotopic signal of phenylalanine in the fungus‐colonized plates remained constant despite increasing consumption of the detrital resource (linear regression: slope = 0, *F*_1,5_ = 0.404; *p *=* *.397, *R* ^2^ = .183). The bulk ^15^N isotopic signal of the fungus‐colonized detritus also remained constant over the same consumption gradient (linear regression: slope = 0, *F*_1,8_ = 0.257; *p *=* *.628, *R* ^2^ = .035). For bacteria, the ^15^N isotopic signal of glutamic acid in the detrital masses increased predictably across the consumption gradient (regression equation: *y *=* *0.571*x* + 4.96, *R* ^2^ = 0.90, *F*_1,5_ = 6.75; *p *=* *.003; Figure 3d), while the isotopic signal of phenylalanine remained constant (linear regression: slope = 0, *F*_1,5_ = 0.182; *p *=* *.691, *R* ^2^ = .044). The bulk ^15^N isotopic signal of the bacteria‐colonized detritus also remained constant over the consumption gradient (linear regression: slope = 0, *F*_1,8_ = 2.09; *p *=* *.192, *R* ^2^ = .23; Figure 3d). All raw data from this trial are contained within Table S1.

### 3.4 Field‐collected animal specimens

Four animal taxa known to be detritivorous (*D. suzukii*, Collembola, *Lumbricus*, Scolytidae) were collected in natural ecosystems and curated for isotopic analyses. Mean (±1 *SE*) trophic positions of fruit flies (*D. suzukii*), springtails (Collembola), earthworms (Lumbricidae), and bark beetles (Scolytinae: Curculionidae) were, respectively, 2.54 ± 0.15 (*N *=* *9), 2.43 ± 0.04 (*N *=* *3), 2.47 ± 0.07 (*N *=* *3), and 2.86 ± 0.05 (*N *=* *3). All means were significantly different (one‐sample *t* test: *p *<* *.05) from their nearest integer value.

## 4 Discussion

While the primacy of microbes within the empire of heterotrophy has been well‐documented (Coleman, 1996; Hagen et al., 2012; Hall & Meyer, 1998; Moore & de Ruiter, 2012; Peterson & Luxton, 1982), the nature and degree to which microbes may reconfigure the trophic identities of carnivore and omnivore groups has remained unresolved. This means that the trophic positions of the single most abundant, massive, and ubiquitous trophic group (detritivorous microbes) have not been measured with known, high accuracy. In our study, we show that the presence of detritivorous bacteria and fungi in brown food chains elevates significantly the trophic positions of detritivorous fauna, including the detrital complex, itself.

### 4.1 Hypothesis 1: Trophic inflation of detrital complexes

We examined brown food webs as trophic hierarchies, and within this framework, measured the degree of trophic inflation caused by bacteria and fungi in a given detrital mass. The microbes were allowed to consume detritus, and in the process, became enmeshed within it, creating a detrital complex. These complexes exhibited marked inflation in trophic position (relative to uncolonized detritus), bringing their trophic positions up to distinctly noninteger positions (Figure 2). The degree of trophic inflation ranged from 60% to 71%, suggesting that most of the detrital proteins had been converted into microbial proteins. Similarly, the carrion that was colonized and consumed by microbes exhibited an increase of 0.60 trophic units (trophic position of carrion with microbes present: 2.7; without microbes: 2.1). In nature, detrital complexes are, functionally, microcosms of the larger food web, replete with basal resources and multiple trophic levels (Bardgett & Cook, 1998; Bengtsson et al., 1996; Moore et al., 1988). It is not surprising, then, that our detrital complexes were significantly elevated in trophic position. Whether plant or animal‐based detritus, such inflation is driven specifically by selective enrichment of ^15^N concentrations within certain amino acid pools, such as glutamic acid (Chikaraishi et al., 2009). The trophic positions of the respective detrital complexes reflected the fact that detritus was converted into bacterial or fungal biomass, elevating the trophic position of the entire complex, but only to the extent that the microbial biomass had displaced detrital biomass. These findings represent the first evidence that microbial “meat” alone can significantly alter the trophic position of the detrital complex, and that the magnitude of this phenomenon is consistent for both plant and animal detritus.

### 4.2 Hypothesis 2: Mechanisms of microbe‐mediated trophic inflation

Our data showed that with increasing bacterial or fungal growth, the ^15^N concentration of glutamic acid within the detrital complex increasingly enriched across a broad consumption gradient (Figure 3). Conversely, the ^15^N concentrations of phenylalanine and bulk‐N remained unchanged across this same gradient. The fact that glutamic acid exhibited significant ^15^N‐enrichment while phenylalanine did not is very typical of these two amino acid pools given their respective metabolic pathways and fractionation tendencies. Unlike phenylalanine, glutamic acid metabolism in consumers involves de‐ and transamination reactions, leading to high ^15^N enrichment with each trophic transfer. (Chikaraishi et al., 2007, 2009; Steffan, Chikaraishi, et al., 2013; Steffan et al., 2015). The unchanged isotopic signal in the bulk‐N samples was also expected because as certain compounds become enriched in ^15^N, the waste products of the consumer become depleted in ^15^N. Bulk N analysis reveals that ^15^N was neither gained nor lost from the detrital complex, even while certain compounds like glutamic acid were effectively stockpiled with ^15^N. In this way, specific compounds within a sample can enrich in ^15^N while the overall ^15^N concentration remains unchanged. Bulk analysis of N includes all nitrogenous compounds, whether organic or inorganic; hence, the ^15^N‐depleted waste products are mixed with the enriched amino acid pools. Similar to their animal counterparts, microbes assimilate key compounds, respire, and eliminate wastes, thereby mineralizing key elements for autotrophic organisms (Kaspari & Yanoviak, 2009). Inorganic wastes are readily rinsed, diluted, or diffused away from the immediate microhabitat of an organism (Pauli et al., 2014). As inorganic wastes with depleted ^15^N diffuse away from an organism, the enriched ^15^N within organismal tissues is retained. This helps to explain how ^15^N often is found to be enriched within soil communities (Hyodo et al., 2008; Ponsard & Arditi, 2000; Tayasu & Hyodo, 2010). Over time, detritus and detritivores are repeatedly consumed, and with each trophic transfer, ^15^N concentrations become enriched within specific amino acid pools of the consumers.

In our petri dish experiments, we created a gradient of microbial consumption that was independent of isotopic concentration. By measuring the extent (%) of microbial consumption of the provisioned detritus (i.e., the media within the petri dishes), we could correlate trophic inflation of the detrital complex with microbial consumption of the detrital substrate (Figure 3). The strong relationship we observed provided evidence that microbes, like their metazoan counterparts (Chikaraishi et al., 2009; Steffan, Chikaraishi, et al., 2013; Steffan et al., 2015), increasingly stockpile ^15^N in the glutamic acid pool, while leaving phenylalanine unchanged. Indeed, across all of these experiments, we observed significant ^15^N‐enrichment within the glutamic acid pools of bacteria‐ and fungi‐colonized detritus (Appendix S1, Table S1). Such compound‐specific ^15^N‐enrichment derives from the transamination of amino acids within the consumer, which fractionates ^15^N as C‐N bonds are cleaved and reformed during the assembly of macromolecules within the consumer (Chikaraishi et al., 2007, 2009). The enrichment of ^15^N within detrital complexes therefore occurs almost entirely within the living components of the complex, as opposed to in the nonliving organic matter and inorganic compounds. Our findings show that as microbial populations grow, they increasingly convert detrital compounds into microbial biomass, effectively displacing the nonliving proportion with living biomass, stockpiling ^15^N within certain amino acid pools. This enriches ^15^N concentrations in select compounds such as glutamic acid (Chikaraishi et al., 2009; Steffan et al., 2015), thereby inflating the trophic position of the entire detrital complex. Microbes literally insert themselves into the food chain, and their impacts are both measurable and significant. The trophic inflation we have reported here reveals the extent to which bacteria and fungi can transform simple detritus into a trophically dynamic complex.

### 4.3 Hypothesis 3: Microbial impacts propagate up the food chain

Microbe‐mediated trophic inflation propagated “up” the hierarchy, directly influencing the trophic identities of detritivorous macrofauna. The fauna in our experiments (i.e., fish, beetles, and moths) exhibited predictably elevated trophic positions (approximately one trophic level above their diets), whether feeding within green or brown food chains (Fig. S1 provides a schematic representation of all the diet‐consumer pairings). When our consumers fed on plant detritus without microbial colonies, their trophic positions clearly registered as herbivores, and when feeding strictly on herbivore biomass, their trophic positions were again shown to be very near their known trophic position. Macrofauna that had fed on bacteria‐ or fungus‐colonized detritus registered distinctly noninteger trophic positions (2.79 ± 0.034 for plant‐based detritus; 3.70 ± 0.012 for carrion‐feeders). These noninteger trophic positions were consistently observed and were predicted based on trophic position measurements of their respective diets (Figure 2). These findings represent the first evidence of detritivorous microbes governing animal trophic position.

### 4.4 Wild, detritivorous fauna

The trophic positions of the free‐roaming animals in our study corroborate our controlled‐feeding experiments. All wild specimens registered intermediate, noninteger trophic positions (2 < *x *<* *3). Strict herbivores should register at trophic position ~2, and strict carnivores (consuming only herbivores) would register at position ~3. Given that the detritivorous animals in our study are known to feed on plant material, the intermediate trophic positions we observed represent strong evidence that these fauna had consumed both autotrophic and heterotrophic biomass. For example, the spotted‐wing drosophila fruit fly, *D. suzukii*, is known to attack fresh fruit, but there is gathering evidence that these flies may preferentially lay eggs within damaged fruit that are in various stages of decay (Steffan, Lee, et al., 2013). Decaying fruit commonly harbor yeasts, and it has long been known that *Drosophila* species are important vectors of yeasts in forest ecosystems (Gilbert, 1980). Recently, the yeast gene (isolated from *Saccharomyces cerevisiae*) responsible for a “fungus aroma” was isolated, and *Drosophila* flies were shown to be attracted only to yeast bearing the aroma gene (Christiaens et al., 2014). After alighting on the yeast substrate, flies readily acquired the yeast cells on their tibiae, allowing them to transport the yeast to new substrates. It is likely that in the field, egg‐laying *Drosophila* would readily inoculate new substrates with yeast cells, which would allow the yeasts to develop alongside fly larvae within host material. Our trophic data provide evidence that *D. suzukii* consumes significant quantities of nonplant protein, such as those deriving from yeasts.

Earthworms and springtails (Figure 1b) are widely known to be detritivorous and ubiquitous in most terrestrial ecosystems (Bardgett & Cook, 1998; Moore & de Ruiter, 2012). In temperate grasslands, springtails tend to be the numerically dominant group, while earthworms represent much greater total biomass (Bardgett & Cook, 1998). Our trophic measurements quantify the degree to which the earthworm and springtail specimens in our study had indulged in carnivory. Both were near trophic position ~2.5, indicating that approximately half of their protein had derived from autotrophic biomass, and the other half, heterotrophic biomass. This suggests that these hyper‐abundant, omnipresent detritivores are distinctly omnivorous, confirming past observations that earthworms and springtails often capture and consume microbial prey (Chahartaghi et al., 2005; van der Heijden et al., 2008). Importantly, our data quantify the degree to which earthworms and springtails rely on nonplant resources in their diet. Because these common detritivores represent a significant prey base for many predators, microbe‐derived trophic inflation will tend to propagate up the food chain, shaping the identities of innumerable macrofauna.

Bark beetle species have long been known to harbor fungal symbionts that are released into host trees as the female beetles deposit their eggs (Paine, Raffa, & Harrington, 1997). The fungus spreads rapidly through the tree cambium, and the beetle larvae follow close behind, feeding on the fungus‐colonized tree tissues. The relatively high trophic position we observed in our bark beetle specimens (~2.8) indicates that these beetles had eaten more fungal proteins than plant proteins.

### 4.5 Implications

Using amino acid isotopic analysis of microbe‐colonized detritus, we have captured the *microbe effect* within brown food chains, demonstrating that faunal trophic identity is a predictable function of microbivory. This establishes an empirical, ecologically portable framework with which to interpret the trophic tendencies of myriad detritivore species. Given that virtually all detritivorous fauna are, to some extent, microbivores and that these fauna represent food for countless carnivore species (Bengtsson et al., 1996; Digel et al., 2014; Moore et al., 1988), the potential for microbial trophic identities to influence those of higher‐order consumers is immense. The fact that all the field‐collected detritivores in our study exhibited noninteger trophic positions suggests that microbe‐mediated trophic inflation is a pervasive phenomenon. Mounting evidence suggests that many generalist omnivore and carnivore species do bear the “fingerprints” of detrital resources (Crotty et al., 2011; Hagen et al., 2012; Haraguchi, Uchida, Shibata, & Tayasu, 2013; Hyodo et al., 2014; Larsen, Taylor, Leigh, & O'Brien, 2009; Newsome, Fogel, Kelly, & Martínez del Rio, 2011; Pollierer et al., 2012). For such fauna, we show that detritivory is, functionally, omnivory. Indeed, detritivorous fauna are intraguild predators (Digel et al., 2014), and our data have quantified the extent to which fauna may prey upon their microbial competitors. With a unified macro‐ and microbiome in trophic ecology, measurement of organismal trophic position, and functional diversity can be rendered more comprehensively.

## Conflict of Interest

None declared.
