# Supplementary material for: Unpacking brown food‐webs: Animal trophic identity reflects rampant microbivory
Source: Ecol Evol. 2017 Apr 9;7(10):3532–41. doi: 10.1002/ece3.2951 (PMC5433990; doi:10.1002/ece3.2951)
Supplement: Supplementary file 1 [file ECE3-7-3532-s001.docx]

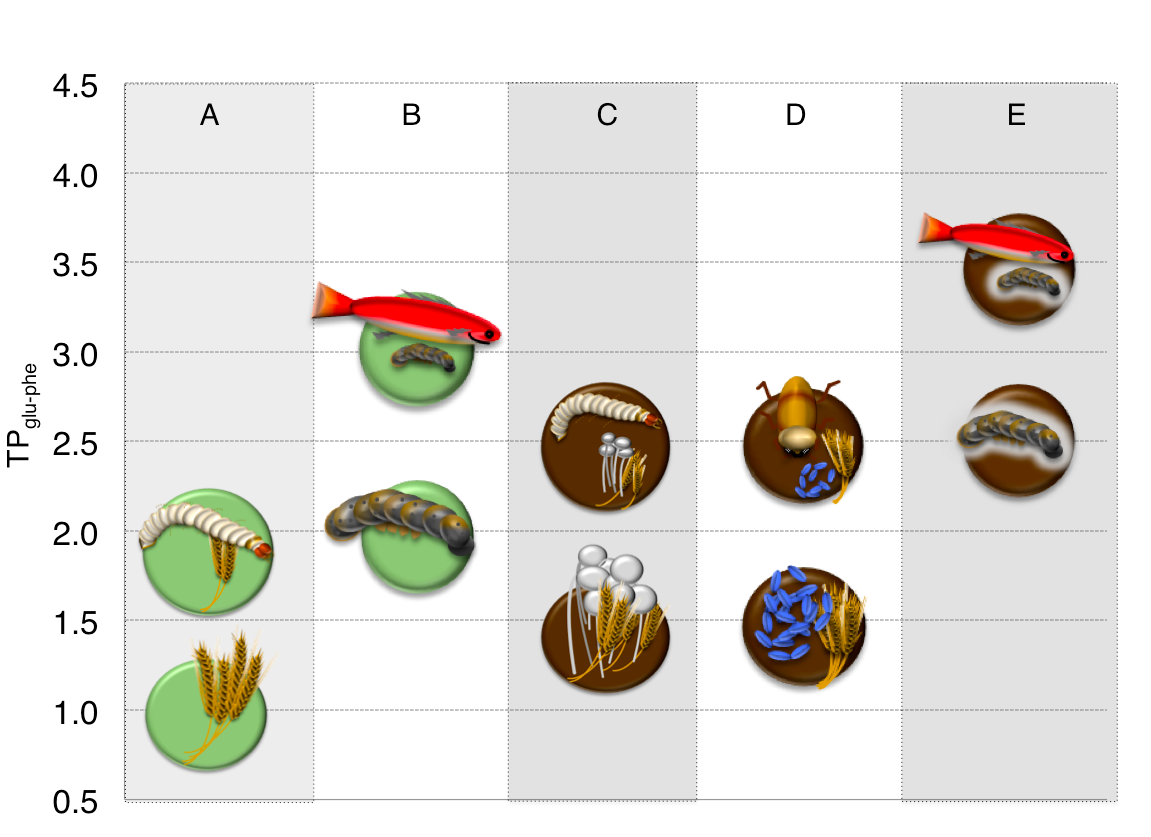


**Figure S1.** Schematic representation of expected trophic positions (ETP) of diet-consumer pairs used in the laboratory-based controlled feeding study. Green circles represent ‘grazer’ food-chains, and brown circles represent ‘detrital’ food-chains. Column A: Green food-chain (strict herbivory), in which the soy-wheat diet (ETP ~ 1.0) was consumed by pantry moth larvae (ETP ~ 2.0). Column B: Green food-chain (strict carnivory), in which fall armyworm larvae were the diet (ETP ~ 2.0), and the consumers were common guppies (ETP ~ 3.0).

Column C: Brown food-chain (plant-based detritivory), in which the diet was a fungus-colonized soy-wheat blend (1.0 < ETP < 2.0), and the consumers were pantry moth larvae (2.0 < ETP < 3.0). Column D: Brown food-chain (plant-based detritivory), in which the diet was a bacteria-colonized soy-wheat blend (1.0 < ETP < 2.0), and the consumers were red flour beetles (2.0 < ETP < 3.0).

Column E: Brown food-chain (carrion feeding), in which the diet was fungus-colonized fall armyworm larvae (2.0 < ETP < 3.0), and the consumers were common guppies (3.0 < ETP < 4.0).
