# Supplementary material for: Unpacking brown food‐webs: Animal trophic identity reflects rampant microbivory
Source: Ecol Evol. 2017 Apr 9;7(10):3532–41. doi: 10.1002/ece3.2951 (PMC5433990; doi:10.1002/ece3.2951)
Supplement: Supplementary file 2 [file ECE3-7-3532-s002.docx]

**Table S1.** Bulk- and compound-specific δ^15^N signals of detrital complexes, cultured over time. The fungus, *Flammulina velutipes,* was cultured on potato dextrose agar (PDA); the bacterium, *Streptomyces*, was cultured on yeast extract maltose agar (YEMA). Both cultures were measured at days 0, 7 and 14.

|  |  | Bulk ^15^N (‰) | | | Mean | σ |  | ^15^N_Glu_(‰) | | Mean | σ |  | ^15^N_Phe_(‰) | | Mean | σ |
| --- | --- | --- | --- | --- | --- | --- | --- | --- | --- | --- | --- | --- | --- | --- | --- | --- |
|  |  | 1 | 2 | 3 |  |  |  | 1 | 2 |  |  |  | 1 | 2 |  |  |
| Fungi and PDA | Day 0 | -0.56 | -0.85 | -0.79 | -0.73 | 0.15 |  | 2.58 | 2.75 | 2.67 | 0.12 |  | 0.16 | 0.36 | 0.26 | 0.15 |
|  | Day 7 | -0.65 | -1.23 | -0.72 | -0.87 | 0.32 |  | 3.16 | 3.41 | 3.29 | 0.18 |  | -0.84 | -0.32 | -0.58 | 0.37 |
|  | Day 14 | -0.69 | -1.06 | -0.71 | -0.82 | 0.21 |  | 5.80 | 6.32 | 6.06 | 0.37 |  | 0.27 | 1.12 | 0.69 | 0.60 |
| Bacteria and YEMA | Day 0 | -0.66 | -0.76 | -0.53 | -0.65 | 0.12 |  | 5.44 | 5.09 | 5.26 | 0.25 |  | 2.99 | 3.08 | 3.04 | 0.07 |
|  | Day 7 | -0.36 | -0.38 | -0.32 | -0.35 | 0.03 |  | 5.93 | 5.77 | 5.85 | 0.12 |  | 3.45 | 3.26 | 3.35 | 0.13 |
|  | Day 14 | -0.27 | -0.44 | -0.65 | -0.46 | 0.19 |  | 7.65 | 8.04 | 7.84 | 0.28 |  | 3.37 | 3.02 | 3.20 | 0.25 |
| PDA alone | Day 0 | -0.70 | -1.10 |  | -0.90 | 0.28 |  | 2.46 |  |  |  |  | 0.05 |  |  |  |
|  | Day 7 | -0.99 | -1.28 |  | -1.14 | 0.21 |  | 2.00 |  |  |  |  | -0.28 |  |  |  |
|  | Day 14 | -1.10 | -0.57 |  | -0.83 | 0.37 |  | 2.26 |  |  |  |  | -0.25 |  |  |  |
| YEMA alone | Day 0 | -0.60 | -0.94 |  | -0.77 | 0.24 |  | 5.35 |  |  |  |  | 3.12 |  |  |  |
|  | Day 7 | -0.81 | 0.06 |  | -0.37 | 0.62 |  | 5.27 |  |  |  |  | 2.96 |  |  |  |
|  | Day 14 | -0.99 | -0.73 |  | -0.86 | 0.18 |  | 4.98 |  |  |  |  | 2.91 |  |  |  |
