# Supplementary material for: Unpacking brown food‐webs: Animal trophic identity reflects rampant microbivory
Source: Ecol Evol. 2017 Apr 9;7(10):3532–41. doi: 10.1002/ece3.2951 (PMC5433990; doi:10.1002/ece3.2951)
Supplement: Supplementary file 3 [file ECE3-7-3532-s003.docx]

**Unpacking brown food-webs: Animal trophic identity reflects rampant microbivory**

(Steffan, Chikaraishi, Dharampal, Pauli, Guédot, & Ohkouchi)

The following information is provided in support of an article published in Ecology & Evolution.

**Supplementary Information**

Materials and Methods

Stable nitrogen isotopic compositions of amino acids were determined by gas chromatography-combustion-isotope ratio mass spectrometry. The abundances of 8-11 amino acids (usually alanine, glycine, valine, leucine, isoleucine, threonine, aspartic acid, serine, methionine, glutamic acid, and phenylalanine) were quantified by comparisons between the peak areas of NPD chromatograms external amino acid references.

The carrier gas (He) flow rate was controlled using a constant flow mode at 1.4 ml min^-1^. Reproducibility of isotopic measurement was assessed using reference mixtures of 9 amino acids with known δ^15^N values (ranging from –25.9‰ to +45.6‰, Indiana University, SI Science Co.), which were analyzed after every 5-8 sample-runs. The isotopic compositions of amino acids in samples were expressed relative to atmospheric nitrogen (N_2_) on scales normalized to known δ^15^N values of reference amino acids.

The respective accuracy and precision for the reference mixtures were 0.0‰ (mean departure from a known ^15^N standard) and 0.4-0.7‰ (1σ) for sample sizes ≥ 1.0 nmol N. The δ^15^N values of alanine, glycine, valine, leucine, isoleucine, glutamic acid, and phenylalanine were determined for all samples based on the S/N ratio ≥ 20, with baseline separation on the chromatogram.

Bulk-N analyses were conducted by the Stable Isotope Core Laboratory at the University of California-Davis. The δ^15^N isotopic ratio for every sample was reported using standard notation, in which *δ*^15^N = [(*R*_sample_/*R*_standard_) – 1)] × 1,000. Here, *R*_sample_ represents the ^15^N:^14^N ratio of a sample, and *R*_standard_ represents the ^15^N:^14^N ratio of atmospheric nitrogen (N_2_). The unit of measurement for *δ*^15^N is per-mil (‰).

**Dataset S1.** Raw data (δ^15^N ‰) for amino acids extracted from the soy-wheat diet.

| Soy-wheat blend (diet) | | Samples analysed (units: δ^15^N ‰) | | |  | Parameters | |
| --- | --- | --- | --- | --- | --- | --- | --- |
|  |  | 1 | 2 | 3 |  | mean | σ |
| Amino acids | Alanine | -4.44 | -4.21 | -4.95 |  | -4.53 | 0.38 |
|  | Glycine | -4.44 | -5.47 | -4.60 |  | -4.84 | 0.56 |
|  | Valine | -4.25 | -4.83 | -5.20 |  | -4.76 | 0.48 |
|  | Leucine | -2.70 | -3.08 | -2.85 |  | -2.88 | 0.19 |
|  | Isoleucine | -4.31 | -4.54 | -4.29 |  | -4.38 | 0.14 |
|  | Proline | -4.10 | -3.94 | -4.86 |  | -4.30 | 0.49 |
|  | Aspartic acid +Threonine | -6.02 | -5.26 | -5.79 |  | -5.69 | 0.39 |
|  | Glutamic acid | -4.45 | -4.43 | -3.77 |  | -4.22 | 0.39 |
|  | Phenylalanine | 2.75 | 3.02 | 3.29 |  | 3.02 | 0.27 |

**Dataset S2.** Raw data (δ^15^N ‰) for amino acids extracted from enoki fungus-colonised (*Flammulina velutipes)* soy-wheat diet.

| Microbe-colonised soy-wheat (diet) | | Samples analysed (units: δ^15^N ‰) | | |  | Parameters | |
| --- | --- | --- | --- | --- | --- | --- | --- |
|  |  | 1 | 2 | 3 |  | mean | σ |
| Amino acids | Alanine | 0.72 | 0.35 | -0.07 |  | 0.33 | 0.40 |
|  | Glycine | -2.62 | -1.59 | -1.36 |  | -1.86 | 0.67 |
|  | Valine | 1.24 | 0.54 | 0.87 |  | 0.88 | 0.35 |
|  | Leucine | 0.59 | 1.29 | 0.39 |  | 0.75 | 0.47 |
|  | Isoleucine | -0.47 | -1.09 | -1.15 |  | -0.91 | 0.38 |
|  | Aspartic acid +Threonine | -0.05 | 0.38 | 0.08 |  | 0.13 | 0.22 |
|  | Glutamic acid | 0.95 | 1.13 | 1.12 |  | 1.07 | 0.10 |
|  | Phenylalanine | 3.22 | 2.83 | 2.79 |  | 2.95 | 0.24 |

**Dataset S3.** Raw data (δ^15^N ‰) for amino acids extracted from the bacteria-colonised (*Bacillus subtilis)* soy-wheat diet.

| Microbe-colonised soy-wheat (diet) | | Samples analysed (units: δ^15^N ‰) | | |  | Parameters | |
| --- | --- | --- | --- | --- | --- | --- | --- |
|  |  | 1 | 2 | 3 |  | mean | σ |
| Amino acids | Alanine | -0.42 | -0.32 | -0.31 |  | -0.35 | 0.06 |
|  | Glycine | -2.92 | -3.47 | -3.20 |  | -3.20 | 0.28 |
|  | Valine | 5.31 | 1.39 | 1.59 |  | 2.76 | 2.21 |
|  | Leucine | -1.41 | -0.98 | -1.59 |  | -1.33 | 0.31 |
|  | Isoleucine | 1.54 | 1.05 | 1.25 |  | 1.28 | 0.24 |
|  | Aspartic acid +Threonine | -0.76 | -1.81 | -1.48 |  | -1.35 | 0.54 |
|  | Glutamic acid | 1.20 | 0.74 | 1.07 |  | 1.00 | 0.24 |
|  | Phenylalanine | 3.96 | 3.68 | 3.36 |  | 3.67 | 0.30 |

**Dataset S4.** Raw data (δ^15^N ‰) for amino acids extracted from the larval fall armyworm (*Spodoptera frugiperda)* diet.

| Herbivorous insect (diet) | | Samples analysed (units: δ^15^N ‰) | | |  | Parameters | |
| --- | --- | --- | --- | --- | --- | --- | --- |
|  |  | 1 | 2 | 3 |  | mean | σ |
| Amino acids | Alanine | 5.52 | 5.05 | 5.44 |  | 5.34 | 0.25 |
|  | Glycine | 0.47 | 0.48 | 1.08 |  | 0.68 | 0.35 |
|  | Valine | 3.36 | 3.64 | 3.97 |  | 3.65 | 0.30 |
|  | Leucine | 2.50 | 2.62 | 2.19 |  | 2.44 | 0.22 |
|  | Isoleucine | 5.38 | 4.93 | 5.10 |  | 5.14 | 0.23 |
|  | Proline | 4.86 | 5.15 | 4.61 |  | 4.87 | 0.27 |
|  | Aspartic acid +Threonine | 3.71 | 4.71 | 4.59 |  | 4.34 | 0.55 |
|  | Glutamic acid | 5.02 | 5.89 | 5.88 |  | 5.60 | 0.50 |
|  | Phenylalanine | 5.49 | 5.62 | 5.26 |  | 5.46 | 0.18 |

**Dataset S5.** Raw data (δ^15^N ‰) for amino acids extracted from the fungus-colonised (*Beauveria bassiana*) fall armyworm diet.

| Microbe-colonised herbivorous insect (diet) | | Samples analysed (units: δ^15^N ‰) | | |  | Parameters | |
| --- | --- | --- | --- | --- | --- | --- | --- |
|  |  | 1 | 2 | 3 |  | mean | σ |
| Amino acids | Alanine | 8.5 | 8.3 | 8.2 |  | 8.34 | 0.17 |
|  | Glycine | 1.3 | 1.6 | 1.4 |  | 1.44 | 0.15 |
|  | Valine | 9.6 | 9.6 | 9.4 |  | 9.54 | 0.08 |
|  | Leucine | 8.1 | 6.9 | 7.3 |  | 7.43 | 0.62 |
|  | Isoleucine | 6.1 | 5.5 | 7.1 |  | 6.22 | 0.84 |
|  | Proline | 9.9 | 9.6 | 9.6 |  | 9.71 | 0.15 |
|  | Aspartic acid +Threonine | 4.4 | 3.3 | 4.7 |  | 4.15 | 0.76 |
|  | Glutamic acid | 10.6 | 10.5 | 10.7 |  | 10.58 | 0.11 |
|  | Phenylalanine | 6.1 | 5.9 | 5.9 |  | 5.94 | 0.15 |

**Dataset S6.** Raw data (δ^15^N ‰) for amino acids extracted from the pantry moth, Plodia (*Plodia interpunctella*) cultured on the soy-wheat diet.

| Herbivorous insect (consumer) | | Samples analysed (units: δ^15^N ‰) | | |  | Parameters | |
| --- | --- | --- | --- | --- | --- | --- | --- |
|  |  | 1 | 2 | 3 |  | mean | σ |
| Amino acids | Alanine | 3.70 | 3.25 | 3.41 |  | 3.46 | 0.23 |
|  | Glycine | -0.90 | -0.40 | -0.96 |  | -0.75 | 0.31 |
|  | Valine | 4.24 | 4.71 | 4.44 |  | 4.46 | 0.23 |
|  | Leucine | 1.08 | 0.69 | 0.57 |  | 0.78 | 0.27 |
|  | Isoleucine | 2.83 | 3.06 | 2.71 |  | 2.86 | 0.18 |
|  | Proline | 2.91 | 2.48 | 2.30 |  | 2.56 | 0.32 |
|  | Aspartic acid +Threonine | 1.60 | 1.44 | 1.07 |  | 1.37 | 0.27 |
|  | Serine |  | 5.60 |  |  | 5.60 |  |
|  | Glutamic acid | 3.77 | 4.31 | 4.81 |  | 4.30 | 0.52 |
|  | Phenylalanine | 3.47 | 3.44 | 3.61 |  | 3.51 | 0.09 |

**Dataset S7.** Raw data (δ^15^N ‰) for amino acids extracted from the pantry moth, Plodia (*Plodia interpunctella*) cultured on the enoki fungi-colonised soy-wheat diet.

| Insect consumer of microbe-colonised plant detritus | | Samples analysed (units: δ^15^N ‰) | | |  | Parameters | |
| --- | --- | --- | --- | --- | --- | --- | --- |
|  |  | 1 | 2 | 3 |  | mean | σ |
| Amino acids | Alanine | 8.43 | 9.42 | 8.44 |  | 8.76 | 0.57 |
|  | Glycine | 3.68 | 3.35 | 4.18 |  | 3.74 | 0.42 |
|  | Valine | 10.94 | 10.66 | 10.66 |  | 10.75 | 0.16 |
|  | Leucine | 5.87 | 4.39 | 4.75 |  | 5.00 | 0.77 |
|  | Isoleucine | 7.24 | 7.22 | 6.34 |  | 6.93 | 0.51 |
|  | Aspartic acid +Threonine | 7.31 | 8.26 | 7.45 |  | 7.67 | 0.51 |
|  | Serine |  | 9.13 |  |  | 9.13 |  |
|  | Glutamic acid | 10.44 | 10.50 | 9.79 |  | 10.25 | 0.39 |
|  | Phenylalanine | 3.41 | 3.75 | 4.20 |  | 3.78 | 0.40 |

**Dataset S8.** Raw data (δ^15^N ‰) for amino acids extracted from the red flour beetle (*Tribolium castaneum*) cultured on the bacteria-colonised soy-wheat diet.

| Insect consumer of microbe-colonised plant detritus | | Samples analysed (units: δ^15^N ‰) | | |  | Parameters | |
| --- | --- | --- | --- | --- | --- | --- | --- |
|  |  | 1 | 2 | 3 |  | mean | σ |
| Amino acids | Alanine | 5.5 | 5.3 | 5.4 |  | 5.4 | 0.12 |
|  | Glycine | 5.5 | 5.0 | 5.5 |  | 5.3 | 0.28 |
|  | Valine | 8.4 | 8.1 | 7.9 |  | 8.1 | 0.26 |
|  | Leucine | 3.9 | 4.3 | 3.5 |  | 3.9 | 0.42 |
|  | Isoleucine | 6.1 | 5.5 | 6.3 |  | 6.0 | 0.46 |
|  | Proline | 6.5 | 5.9 | 6.3 |  | 6.3 | 0.29 |
|  | Aspartic acid +Threonine | 1.3 | 1.8 | 1.6 |  | 1.6 | 0.28 |
|  | Glutamic acid | 10.3 | 9.6 | 9.5 |  | 9.8 | 0.45 |
|  | Phenylalanine | 3.3 | 3.6 | 3.6 |  | 3.5 | 0.18 |

**Dataset S9.** Raw data (δ^15^N ‰) for amino acids extracted from guppy (*Poecilia reticulata*) cultured on the larval fall armyworm diet.

| Fish consumer of insect detritus | | Samples analysed (units: δ^15^N ‰) | | |  | Parameters | |
| --- | --- | --- | --- | --- | --- | --- | --- |
|  |  | 1 | 2 | 3 |  | mean | σ |
| Amino acids | Alanine | 8.00 | 9.73 | 8.48 |  | 8.7 | 0.89 |
|  | Glycine | 0.46 | 1.90 | 2.48 |  | 1.6 | 1.04 |
|  | Valine | 8.10 | 7.77 | 7.35 |  | 7.7 | 0.37 |
|  | Leucine | 4.68 | 5.23 | 5.30 |  | 5.1 | 0.34 |
|  | Isoleucine | 9.05 | 9.53 | 8.73 |  | 9.1 | 0.40 |
|  | Aspartic acid +Threonine | 7.02 | 8.39 | 8.43 |  | 7.9 | 0.80 |
|  | Glutamic acid | 13.94 | 13.06 | 12.77 |  | 13.3 | 0.61 |
|  | Phenylalanine | 5.85 | 5.53 | 5.75 |  | 5.7 | 0.17 |

**Dataset S10.** Raw data (δ^15^N ‰) for amino acids extracted from guppy (*Poecilia reticulata*) cultured on the fungus-colonised fall armyworm diet.

| Fish consumer of microbe-colonised insect detritus | | Samples analysed (units: δ^15^N ‰) | | |  | Parameters | |
| --- | --- | --- | --- | --- | --- | --- | --- |
|  |  | 1 | 2 | 3 |  | mean | σ |
| Amino acids | Alanine | 14.5 | 8.2 | 13.2 |  | 12.0 | 3.31 |
|  | Glycine | 3.6 | 4.4 | 6.1 |  | 4.7 | 1.30 |
|  | Valine | 14.1 | 13.9 | 13.7 |  | 13.9 | 0.20 |
|  | Leucine | 9.1 | 9.3 | 8.6 |  | 9.0 | 0.37 |
|  | Isoleucine | 11.5 | 11.2 | 12.0 |  | 11.6 | 0.41 |
|  | Aspartic acid +Threonine | 6.1 | 6.7 | 5.7 |  | 6.1 | 0.50 |
|  | Glutamic acid | 18.1 | 18.2 | 18.5 |  | 18.3 | 0.18 |
|  | Phenylalanine | 6.1 | 6.0 | 6.3 |  | 6.2 | 0.17 |
